# Conventional and Eccentric Uses of Crystallographic Databases in Practical Materials Identification Problems

**DOI:** 10.6028/jres.101.029

**Published:** 1996

**Authors:** James A. Kaduk

**Affiliations:** Amoco Corporation, Naperville Analytical Technical Laboratory Services, P.O. Box 3011 MC F-9, Naperville, IL 60566

**Keywords:** Cambridge Structural Database, cobalt pyromellitate, copper aluminum borate, Inorganic Crystal Structure Database, magnesium chloride tetrahydrate, NIST Crystal Data, palladium chloride, potassium aluminum borate, Powder Diffraction File, relational databases, terephthalic acid, vanadium phosphate

## Abstract

The crystallographic databases are powerful and cost-effective tools for solving materials identification problems, both individually and in combination. Examples of the conventional and unconventional use of the databases in solving practical problems involving organic, coordination, and inorganic compounds are provided. The creation and use of fully-relational versions of the Powder Diffraction File and NIST Crystal Data are described.

## 1. Introduction

The seven widely available databases which contain crystallographic information—the Cambridge Structural Database [1], Inorganic Crystal Structure Database [2], Metals Data File [3], Protein Data Bank [4], Nucleic Acid Database [5], NIST Crystal Data [6], and Powder Diffraction File [7]—are powerful and cost-effective tools for solving materials identification problems. They assist in answering the question “What is this?” at levels from fingerprint matching to determining subtle details of the atomic arrangements.

A difficulty in illustrating the practical use of these databases is that most applications are “routine.” A query is posed, and the answer is found, solving the problem. Alternatively, no “hit” may be found, suggesting that the problem may be novel. The rapid solution of problems represents their most important use, and saves enough time to justify the costs of the databases. Just as no one analytical tool solves all problems, no one database yields all the answers. It is often necessary to use these databases in combination to solve a particular problem.

We generally think of the databases as repositories of atom coordinates, but they also contain valuable bibliographic information, and can represent useful entries into the primary literature. They can also broaden our thinking. Knowing what structures are “out there” can result in new insights into what “might be.” The databases provide the raw material and tools for assessing structural similarity qualitatively and quantitatively. They enhance scientific productivity and creativity. I routinely use them to “solve” crystal structures.

I have selected database applications from recent work in my laboratory. These examples represent solutions to scientifically interesting problems, but also serve to illustrate things about the databases themselves. Both explicitly and implicitly I seek to illustrate the strengths and weaknesses of the databases, and to make suggestions for database development. In these examples, I slight the Protein Data Bank, the Nucleic Acid Database, and the Metals Data File, since I am not currently using them as often as the other databases in solving refining and petrochemical problems. I give the Crystal Data Identification File perhaps more than expected prominence, but it is often the database I enter first.

## 2. Organic Compounds

### 2.1 Bis(triphenylsilyl) Peroxide

A sample purported to be triphenylsilylhydroperoxide, (C_6_H_5_)_3_SiOOH, contained a few suitable single crystals. One of these was used to determine the primitive triclinic lattice parameters *a* = 8.779(4), *b* = 9.437(3), *c* = 11.322(5) Å, *α* = 65.74(3), *β* = 89.62(3), and *γ* = 66.17(3)°. The numbers in parentheses are estimated standard deviations resulting from a least-squares refinement of the lattice parameters. A default search of the organic portion of NIST Crystal Data yielded 4 hits:
FORMULA:C14 H22 Cl N3 PdRECORD:786834FORMULA:C36 H30 Ge O2 SiRECORD:802546FORMULA:C36 H30 O2 Si2RECORD:803099FORMULA:C36 H30 O2 Si2RECORD:805973The first can be discarded because the cell angles do not match the observed angles, and because the composition is unlikely given the synthesis. The last three hits correspond to two isostructural hexaphenyl compounds, a bis(silyl) and a germylsilyl compound which crystallize in unit cells very similar to that of the material being examined. The compound was thus identified as bis(triphenylsilyl) peroxide.

An important consideration in such a phase identification is whether a representative sample has been obtained. A single crystal was selected from the sample, with no assurance that it represented the bulk. Most crystallographers have from time to time been victimized by an impurity phase which happens to crystallize much more easily or better than the material of interest.

One way around this potential sampling problem is to measure a powder pattern of the bulk material. The crystal structure of bis(triphenylsilyl) peroxide [8] is contained in the Cambridge Structural Database. The powder pattern calculated from the reported crystal structure matched the experimental pattern well. The single crystal did not represent an impurity phase, and thus the effort to determine the crystal structure was saved.

### 2.2 Isophthalic Acid

Powder patterns of 1,3-benzenedicarboxylic acid match the PDF entry 37-1920, but several weak, low-angle lines are not accounted for by this database pattern, and the match of the relative intensities is not as good as desired. Much time (and thus money) can be spent in trying to identify impurity phases indicated by such “extra” peaks. The powder pattern calculated from the CSD structure [9] explains these weak low-angle lines, and the calculated intensities match well those of the experimental patterns. This example demonstrates that even carefully edited databases may be only as good as the data input to them. Having access to all the crystallographic databases is cost effective; it doesn’t take much wasted time to pay for them.

### 2.3 Terephthalic Acid

**Fig. 1:**
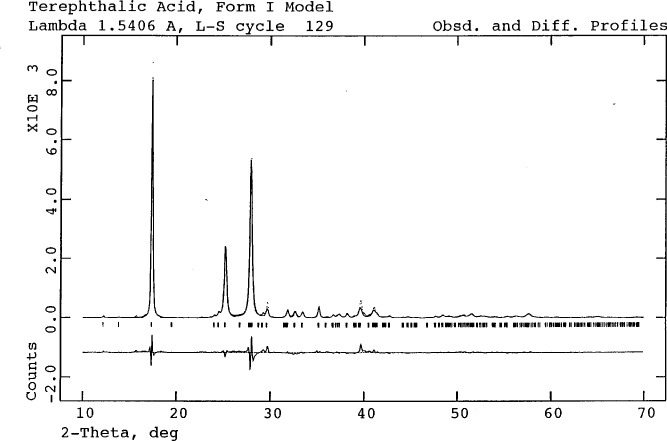
Observed, calculated, and difference powder diffraction patterns of terephthalic acid, using the “Form I” model of Bailey and Brown (Ref. 9). The dots represent the experimental points, and the solid line the calculated pattern. The difference curve is plotted at the same scale as the other patterns. The row of tick marks represents the calculated line positions. The relatively small residuals indicate the present of approximately 25 % of the “Form II” polymorph.

Powder patterns of commercial terephthalic acid (1,4-benzenedicarboxylic acid) agree well with the PDF pattern 31-1916. Rietveld refinements of some patterns using the published structural model [9], however, are unsatisfactory (Fig. 1). The strong peaks exhibit varying degrees of asymmetry, and the fit to the weak lines is poor. The structure corresponding to the PDF entry is Bailey and Brown’s “Form I” [10]. These authors also report the crystal structure of another polymorph, “Form II.” The reported distances and angles for this polymorph cannot be reproduced using the reported coordinates and cell. It is clear that the coordinates of at least one of the atoms are incorrect.

When the CSD is searched for crystal structures of terephthalic acid, it is found that errors in both the coordinates and lattice parameters of Form II were corrected some years later [11]. Using this corrected model, both we and others [12,13] obtain much better agreement between the observed and calculated patterns. Some samples of terephthalic acid consist of mixtures of polymorphs, which can be interconverted. The sample of Fig. 1 contained approximately 25 % of Form II.

The moral here is that the databases are sometimes *better* than original literature! Not infrequently I find that errors in the original literature have been corrected. It is also worth looking at the actual database contents, and not just using a graphical interface. There are valuable comments and notations that can be overlooked when visualizing the structures.

## 3. Coordination Compounds

### 3.1 Cobalt Pyromellitate

A crystalline orange material was isolated from an oxidation of durene (1,2,4,5-tetramethylbenzene) using a homogeneous Co/Mn/Br catalyst system in an acetic acid/water solution. Standard single-crystal techniques indicated a primitive monoclinic unit cell having *a* = 6.545(3), *b* = 9.933(3), *c* = 41.097(17) Å, and *β* = 89.94(3)°. A default search of this cell in NIST Crystal Data yielded no hits. An intensity data set was collected. No systematic absences were observed, consistent with space groups P2, Pm, or P2/m. Attempts to solve the structure were unsuccessful.

A portion of the sample was ground, and mixed with NIST SRM675 (fluorophlogopite) internal standard. Peaks in the powder pattern were located by interactive deconvolution. The corrected positions of 41 peaks yielded a primitive monoclinic cell (Visser ITO [14]; figure of merit = 99.1) having *a* = 6.545, *b* = 9.924, *c* = 6.497 Å, and *β* = 115.45°.

A search of this cell in NIST Crystal Data yielded 5 inorganic and 1 organic hits:
FORMULA:Pb2 Mn2 Si2 O9RECORD:727233FORMULA:Mn2 Pb2 Si2 O9RECORD:727905FORMULA:Pb2 (Mn, Fe)2 Si2 O9RECORD:730552FORMULA:K4 Pb O4RECORD:735661FORMULA:K4 Pb O4RECORD:735674FORMULA:C10 H4 O8 −2 ! H12 Co O6 +2RECORD:253709

The five inorganic hits could be discarded immediately, because the chemistry was not similar to that of this problem. The last hit is the hexaaquacobalt(II) salt of the dianion of pyromellitic acid (1,2,4,5-benzenete-tracarboxylic acid). This chemistry is quite reasonable for a product of this oxidation.

**Fig. 2:**
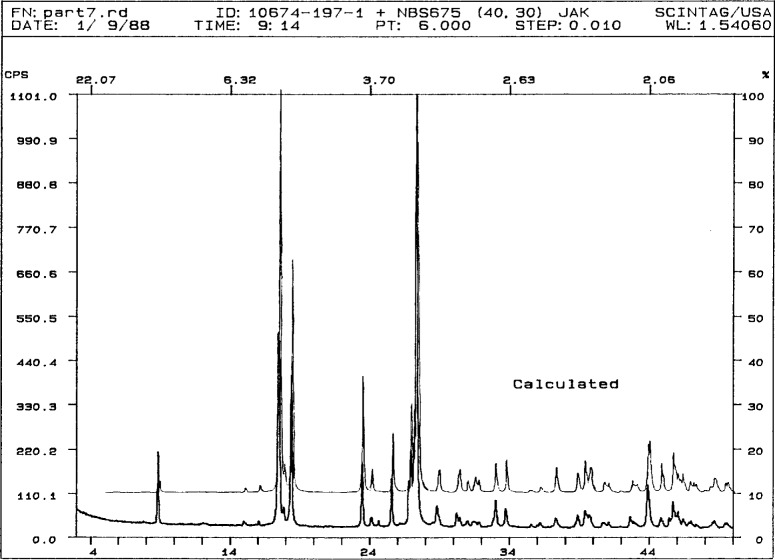
Observed and calculated patterns of hexaaquacobalt(II) dihydrogenpyromellitate.

A search of the CSD for compounds containing a pyromellitate fragment and only Co, C, H, and O yielded two hits: hexaaquacobalt(II) dihydrogen-1,2,4,5-benzenetetracarboxylate [15] (the compound with matching cell) and *catena*((µ^2^-pyromellitato)tetraaquacobalt(II) octahydrate [16]. The powder pattern calculated for the first compound is identical to the observed pattern (Fig. 2), confirming the identification. The calculated pattern is now included in the PDF as entry 45-1707. The second CSD “hit” provides additional insight into the kinds of compounds which might form in such a chemical system.

Crystallization of this hexaaqua compound was unexpected, but sensible in hindsight. Understanding of the oxidation chemistry derived from this phase identification helped rationalize a process patent.

The “single” crystal was apparently a twin. The strategy of grinding a crystal into a powder for a phase identification seems perverse, but is occasionally useful. The volume of the single crystal cell is 2671 Å^3^, 7.01 times larger than the 381 Å^3^ of the cobalt pyromellitate cell. The similarity of the *a* and *b* cell dimensions of the apparent and true cells suggests that there might be some relationship between them.

A search of the original “single crystal” cell in the organic portion of NIST Crystal Data for subcells having as low as 1/9 the initial cell volume yielded 103 hits. This selection set can be limited by the use of chemical constraints. It can be reduced to 36 entries by looking at only compounds which contain C, H, and O—as would be expected from an oxidation reaction. (We chose not to specify the metal atom, since we didn’t know what isostructural complexes might have been characterized.) Among the 36 hits is the cobalt pyromellitate. Although not relevant for the solution of this particular problem, this search illustrates how NIST Crystal Data can be used to search for structural relationships among compounds having apparently dissimilar cells, but cells which are related by a transformation.

### 3.2 Magnesium Ethoxide

The powder pattern of this highly moisture-sensitive material is not in the PDF, and the crystal structure has not yet been reported. A search of NIST Crystal Data for compounds containing only Mg, C, H, and O yielded 66 hits. One of these, 2(C_2_H_5_O^−^)Mg^2+^, corresponds to magnesium ethoxide [17]. The space group is $P\bar{3}m1$, with *a* = 3.10 and *c* = 9.40 Å, but the atom coordinates have never been reported.

The cell, crystal system, and general chemical knowledge make it almost certain that the structure of magnesium ethoxide consists of brucite (Mg(OH)_2_, $P\bar{3}m1$, *a* = 3.1442(7), *c* = 4.777(2) Å) layers in which the hydroxyl protons are replaced by ethyl groups. The observed cell, the brucite structure, and a molecular mechanics program were used to derive carbon atom positions. The powder pattern calculated from this model was a good match to the observed pattern of magnesium ethoxide.

### 3.3 Thiophene Complexes

To provide raw material for computational studies of metal-thiophene complexes related to sulfur removal from naphtha, the CSD was searched for crystal structures containing a thiophene fragment and a Group VIII metal. The 24 hits included complexes of all Group VII metals except Co and Ni. Six different binding modes—monodentate S, bridging S, η^4^ (2,3,4,5), η^2^ (2,3), σ-bonded at 2, and σ-bonded at 3—were observed. Not all of these had been considered in the quantum mechanical calculations. The information in the database broadened our ideas about possible binding modes, and increased our confidence that global minimum energy structures would be found. The efficiency of computational studies is improved when good initial models extracted from the databases are used. Nature is also more clever than we imagine.

## 4. Inorganic Compounds

### 4.1 Potassium Aluminum Borate

During exploration of the K_2_O-Al_2_O_3_-B_2_O_3_ ternary phase diagram, it was discovered that a *black semiconducting amorphous phase* could be formed near the composition 1K_2_O : 1Al_2_O_3_ : 2B_2_O_3_. Only one ternary phase [18], K_3_AlB_8_O_15_, had been reported in this phase diagram. This ternary, and the known binary phases, were located by searching NIST Crystal Data, the Inorganic Crystal Structure Database, and the Powder Diffraction File.

From preparations having compositions near 1K_2_O : 1Al_2_O_3_ : 1B_2_O_3_, a phase with a new powder pattern was synthesized. The composition of the phase was found to be K_2_Al_2_B_2_O_7_. Since this phase is formed near the semiconducting phase in the phase diagram, we hoped that knowledge of its crystal structure would provide some insight into the structure of the amorphous phase and the mechanisms of conductivity.

A search of the experimental pattern against the PDF yielded no plausible isostructural or model compounds. The pattern could be indexed on a very high quality trigonal/hexagonal unit cell having *a* = 8.55800(2) and *c* = 8.45576(3) Å, with no systematic absences. A default search of the inorganic portion of NIST Crystal Data yielded 13 hits. The least-implausibly related materials were Hf_18_Mo_8_Ni_2_O_1.68_ and (Zn,Be)_2_SiO_4_. The space group of the first is reported as P6_3_/mmc, but no information on the structure is available. The second is reported to have space group R3, with “limited” structural information. Neither of these seemed plausible structural models.

When the default search windows were widened, and a subcell search down to 1/4 the volume was carried out, 968 hits were located. Limiting the set to only those compounds containing oxygen reduced the size to 297 hits. Among these were many references to compounds like RbAl(SO_4_)_2_, which has a large cation, an octahedral cation, and two tetrahedral anions in the formula unit. This has the wrong stoichiometry, and we know from NMR that the Al are tetrahedral and the B trigonal. There were also many references to compounds of the type YbAl_3_(BO_3_)_4_. We knew from previous experience that this structure type was not a good model. Equivalent searches on supercells yielded no more-promising models.

It turns out that the stoichiometry of K_2_Al_2_B_2_O_7_ is unusual. A search of the ICSD for formula type ANX = A2B2C2X7 yielded only 9 hits. Among these were three references to Na_2_Zn_2_Si_2_O_7_ and three to Na_2_Mn_2_Si_2_O_7_. These two compounds have the wrong connectivity. Also found was Rb_2_Be_2_Si_2_O_7_ [19]. This compound contains trigonal planar Be and Si_2_O_7_ units. The powder pattern (PDF 29-1081) confirmed that it might be a good model structure.

**Fig. 3:**
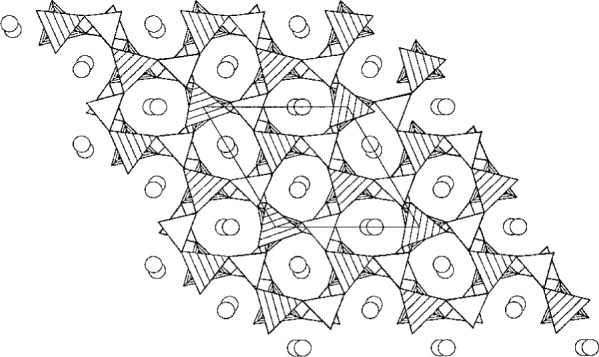
The crystal structure of K_2_Al_2_B_2_O_7_, viewed in projection down the trigonal [001] axis. The open triangles represent the BO_3_ units, and the shaded tetrahedra indicate the AlO_4_ subunits.

Rb_2_Be_2_Si_2_O_7_ crystallizes in P2nn with *a* = 8.92, *b* = 8.32, and *c* = 5.15 Å. It turned out to be easier to solve the structure of K_2_Al_2_B_2_O_7_ *ab initio* from synchrotron powder data than to make all of the necessary coordinate transformations. The space group of K_2_Al_2_B_2_O_7_ is P321. It has a 3-dimensional network structure (Fig. 3) [20], which does indeed have the same framework topology as that of Rb_2_Be_2_Si_2_O_7_. There are small differences in torsion angles, but the compounds are isostructural.

The astute reader will have noticed that only seven of the nine ICSD hits have been discussed. The additional two were Rb_2_Pb_4_O_7_ (which has the wrong connectivity) and K_2_Pb_2_Ge_2_O_7_, which contains trigonal Pb and tetrahedral Ge in Ge_2_O_7_ units. This is not a network, but a layered structure, very similar to that observed for SrAl_2_B_2_O_7_ [21]. The fact that B and Pb could fill similar roles in a structure is a surprise.

In identifying a material or solving and analyzing a crystal structure, we are often interested in locating similar structures. This could mean isostructural materials, or merely compounds related in some way. Our searches of the PDF, NIST CD, ICSD, and other databases are ways of indirectly identifying similar structures. It would be much more efficient if we had better ways of defining infinite inorganic structures, and had qualitative and quantitative measures of structural similarity. My ultimate goal is to do a connectivity search in the ICSD just as we can do in the CSD. Consider this a plea to database designers and developers! For inorganic structures, I have been intrigued by the idea of using overlap integrals of Patterson functions as a measure of structural similarity.

### 4.2 Copper Aluminum Borate

The unusual copper aluminum borate Cu_2_Al_6_B_4_O_17_ is useful as a dehydrogenation catalyst [23]. The average structure (I4/m, *a* = 10.586(1), *c* = 5.688(2) Å) has been known for some time [23], and has been redetermined recently using single-crystal techniques [24]. Structure determination has been hampered by the difficulty of preparing homogeneous materials. Recent advances in sol-gel preparative chemistry [22] have led to the synthesis of uniformly green material, permitting a more-detailed structural study.

**Fig. 4:**
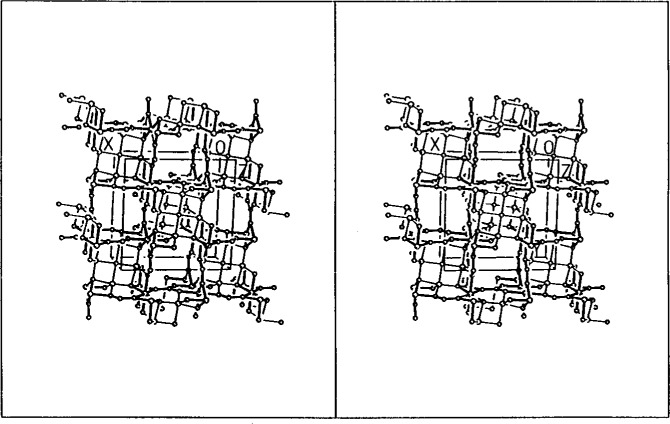
A stereo view of the crystal structure of Cu_2_Al_6_B_4_O_17_. The view is approximately down the tetragonal *c*-axis. The AlO_6_ bonds are highlighted.

The crystal structure (Fig. 4) is made up of edge-sharing chains of octahedral Al atoms parallel to the tetragonal *c*-axis. The AlO_6_ chains are joined in the *a*-and *b*- directions by trigonal planar BO_3_ groups. There is a 5-coordinate site, 50 % occupied each by Cu and Al, which shares a face with the AlO_6_ octahedron. These trigonal bipyramidal sites share equatorial corners at a square planar oxygen, O1.

Trigonal bipyramidal coordination is relatively unusual for both Cu^2+^ and Al^3+^. Difference in typical Cu-O and Al-O distances suggested the possibility that Cu and Al might occupy slightly different positions within the O5 coordination sphere. Attempts to refine such a split-site model using laboratory powder data did not yield improved residuals compared to a unified-site model. To study this site in more detail, we carried out a resonant powder diffraction experiment [25], exploiting the tunability of synchrotron radiation.

The Cu and Al do not occupy different sites, but a common position. The trigonal-bipyramidal Cu1/Al1 site is half occupied each by Cu and Al. The axial distances to two O2, and are long and short (1.998(3) and 1.854(3) Å). Two of the equatorial distances (to O4) are short (1.872(2) Å) and one (to O1) is long (2.038(1) Å). The central Cu1/Al1 site is displaced 0.24 Å from the center of the coordination polyhedron.

The atomic valences, calculated from the sums of bond valences [26], of the Cu and Al are 2.63 and 2.44, far from the nominal values of 2 and 3. The calculated valence of O1 is only 1.54, reflecting the relatively long bonds. These anomalies are indications that the refined structure represents an average.

Analysis of 81 Cu^2+^O_5_ coordination spheres located in the Inorganic Crystal Structure Database indicates that the typical CuO_5_ coordination sphere contains four bonds in the range 1.90 Å–2.05 Å, and one longer bond, averaging 2.2 Å–2.3 Å. The average Cu1 coordination sphere is therefore very unusual, in that all five bonds are shorter than 2.04 Å. The Cu-O2 bond of 1.85 Å is among the shortest Cu-O bonds ever reported.

EXAFS experiments [27] provide evidence for Cu clustering. Each Cu has at least one Cu in the second coordination sphere. This observation, and the appearance of the *F*_obs_ map, suggest a new model for the local structure.

**Fig. 5:**
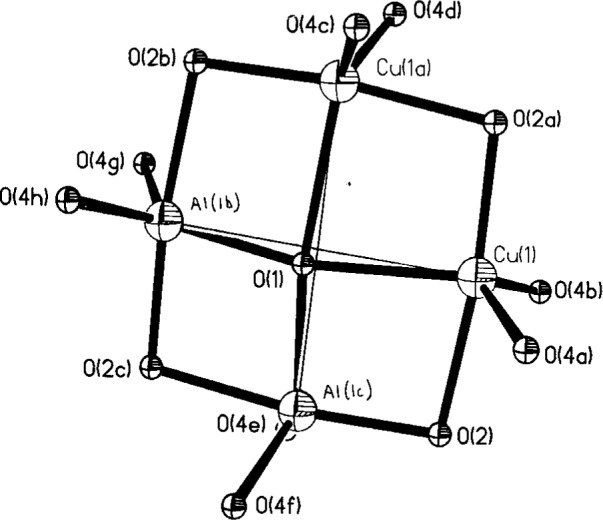
The proposed model for the local environment of the Cu/Al sites in Cu_2_Al_6_B_4_O_17_. The true position of O1 is displaced approximately 0.27 Å from the average position. 50 % probability ellipsoids.

Consider the four 5-coordinate sites surrounding an individual O1. Stoichiometry mandates that there are two Cu and two Al in the average “4-ring” around O1, and that there is only one oxygen in the center of the “4-ring.” If, according to the EXAFS results, the Cu ions occur in “*cis*” pairs, a displacement of the central oxygen away from the two Cu in the *xy* plane would result in two long Cu-O1 bonds and two short Al1-O1 bonds (Fig. 5). A displacement of approximately 0.27 Å along [110] permits the bonding requirements of all atoms to be better-satisfied, is consistent with the EXAFS data, yields comparable residuals to the ordered model for O1, and describes the same average structure. The combination of crystallographic and spectroscopic information has resulted in a new model for the local structure, a model consistent with all observations and with the catalytic properties of this material. The structural insights developed by statistical analysis of database contents were crucial to the development of this model.

### 4.3 Palladium Chloride

To check the suitability of reagent PdCl_2_ as an EXAFS reference material, the powder pattern was measured. The observed pattern matched the PDF pattern 1-228 well enough to confirm the identification. The database pattern did not, however, account for all of the observed lines.

The crystal structure of *α*-PdCl_2_ is included in the ICSD [28]. The PDF entry 1-228 includes the unit cell from this structure determination. The observed relative intensities did not correspond exactly to the database pattern. To determine the source of the discrepancy, the powder pattern was calculated from Wells’ structure. The calculated pattern does *not* correspond to the database pattern.

A second polymorph, *β*-PdCl_2_, which contains isolated Pd_6_Cl_12_ molecules, has been reported [29]. A powder pattern calculated from this structure does not correspond to the observed pattern.

Heating the reagent palladium chloride in a chlorine atmosphere at 500 °C [30] yields a material which matches that calculated from Wells’ structure. A Rietveld refinement of the pattern indicated a few shoulders, best explained by an additional polymorph having the CuCl_2_ structure (PDF entry 35-690) [31]. This structure consists of a different packing of the same chains as in the *α*-PdCl_2_ structure. Including this second phase in the Rietveld refinement improved the fit, but the residuals indicated that some stacking faults were probably present.

This problem illustrates the advantages of having ready access to the databases, but that you can’t believe everything in them! They are also not complete, as we had to resort to the primary literature to locate the phases relevant to this problem. Despite the imperfections, the databases can lead to structural insights, when combined with chemical knowledge.

### 4.4 Vanadium Phosphates

Vanadyl pyrophosphate, (VO)_2_P_2_O_7_, is believed to be the active phase in the air oxidation of butane to produce maleic anhydride. The structure reported in the ICSD [32] contains the ominous warning “coordinates from paper obviously wrong.” In fact, there is a typographical error in the coordinates of O18, but the rest of the asymmetric unit is correct. When the distances and angles are calculated, those within the asymmetric unit are reasonable, but those involving a symmetry transformation are wrong. It turns out that the coordinates correspond not to the reported space group Pca2_1_, but to the alternate setting Pb2_1_a.

Essentially the same structure (also containing errors) was reported by Middlemiss [33]. Recent work by Thompson [34] and by Sleight [35] has provided much better insight into the true structure of this important material. Calculating the distances and angles provides a powerful check on the quality of the structure report, and can enable recovery from errors.

An attempt to prepare single crystals of vanadyl pyrophosphate yielded massive clusters of purplish-black crystals, with a few olive green, orange, and multicolored inclusions. The best match to the powder pattern of the bulk sample was 33-1443, VO(PO_3_)_2_.

To gain insight into the impurity phases present, one of the green inclusions was isolated, and the primitive tetragonal unit cell, having *a* = 6.02(2) and *c* = 4.42(4) Å, was determined using standard single-crystal techniques. A search of the inorganic portion of Crystal Data yielded 6 hits:
FORMULA:(P H4) BrRECORD:292090FORMULA:P H4 BrRECORD:292103FORMULA:V O P O4RECORD:300084FORMULA:V P O4RECORD:300098FORMULA:V P O5RECORD:300112FORMULA:V1.08 P0.92 O5RECORD:302760

The first two can be discarded because the chemistry is not reasonable. The last four correspond to *α*-VOPO_4_, P4/n, *a* = 6.014(7) and *c* = 4.434(2) Å. The similarity of the cell dimensions and the crystal system confirm the identity of the green inclusions as *α*-VOPO_4_. This compound is a quite reasonable byproduct from such a synthesis. The formula of database entry 300098, VPO_4_, is clearly a typographical error.

**Fig. 6:**
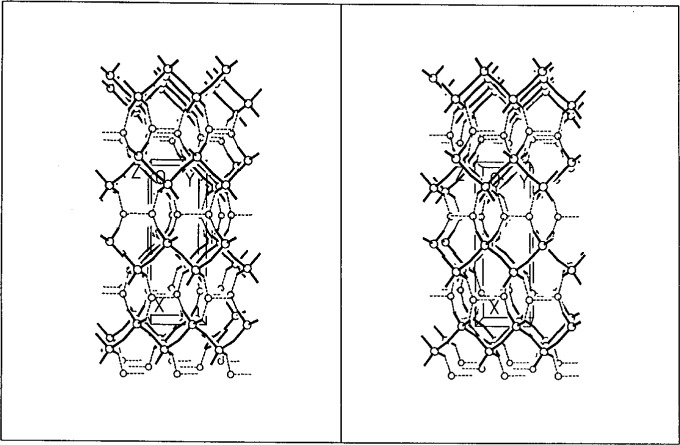
A stereo view of the crystal structure of monoclinic VOP_2_O_6_, viewed down the *c*-axis. The PO_4_ bonds of the polyphosphate chains are represented by dark solid lines, and the VO_5_ coordination spheres by dotted bonds.

A single crystal of the major phase was isolated, and the structure determined using standard techniques. The compound crystallizes in the monoclinic space group I2/a, with *a* = 12.170(2), *b* = 4.1998(13), *c* = 9.573(2) Å, *β* = 92.834(16)°, and *Z* = 4. A search of this cell in the inorganic portion of NIST Crystal Data yielded no hits. The structure is best described as vanadyl polymetaphosphate (Table 1), and consists of infinite corner-sharing PO_4_ polyphosphate chains parallel to the *b*-axis, joined together by square pyramidal VO_5_ polyhedra, sharing basal oxygens with the polyphosphate chains (Fig. 6).

The structure of tetragonal *β*-VOP_2_O_6_ has been reported [36], and essentially the same structure was reported by Middlemiss [33]. The powder pattern calculated from this structure matches neither the PDF entry nor our observed pattern. The powder pattern of VOP_2_O_6_ has also been reported by Bordes and Courtine [37]. Their pattern corresponds neither to the PDF entry nor to the pattern calculated for our monoclinic structure.

All references in the primary literature [33,37–41] which contain any crystallographic information on VP_2_O_7_ refer to the tetragonal cell, but two of them [38,39] also refer to “*α*-VOP_2_O_6_”. We believe that our monoclinic polymorph corresponds to this *α* form. The topologies of the two polymorphs are the same, but the orientations of the chains and vanadyl polyhedra differ. Calculated patterns of the monoclinic and tetragonal polymorphs are now included in the PDF (43-309 and 44-66, respectively).

Although extensive, the databases are not complete. It is not possible to avoid searching the primary literature. Errors are also present. This is an extreme example, since the chemistry of vanadium phosphates is very complicated.

### 4.5 Magnesium Chloride Tetrahydrate

The powder pattern of the preparation of a polypropylene catalyst precursor matched that of MgCl_2_·4H_2_O (1-1210). This PDF entry is the *only* reference in the crystallographic literature to this compound. Since Mg^2+^ is about the same size as a number of divalent first transition series cations, and since many Mg salts are isostructural to those of divalent transition metals, the inorganic portion of NIST Crystal Data was searched for compounds containing only (Fe, Co, Ni, or Zn), Cl, O, and H.

**Fig. 7:**
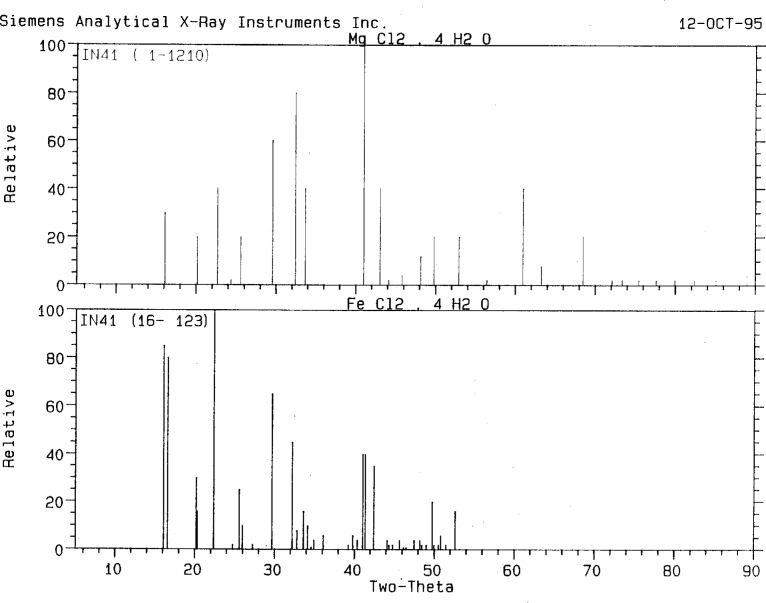
The PDF patterns of MgCl_2_·4H_2_O and FeCl_2_·4H_2_O. The differences in line positions and relative intensities are sufficient to obscure the fact that these compounds are isostructural.

The search was carried out as four separate “only” searches. Among the hits were two structure determinations of FeCl_2_·4H_2_O. One of them was a neutron single crystal study, in which the hydrogen atoms were located. After adjusting the lattice parameters to correspond to the observed peak positions, this model proved good enough to permit a Rietveld refinement of the crystal structure of MgCl_2_·4H_2_O. Both compounds crystallize in P2_1_/n:
Compound*a**b**c**β*(Å)(Å)(Å)(Å)MgCl_2_·4H_2_O5.8966(11)7.2684(7)8.4171(9)110.98(2)FeCl_2_·4H_2_O5.885(3)7.180(6)8.514(4)111.09(2)The powder pattern of FeCl_2_·4H_2_O is present in the PDF (16-123). The differences in the lattice parameters and site occupancies result in differences both in positions and intensities in the powder patterns 1-1210 and 16-123 (Fig. 7), helping to explain why the identification of isostructures was not made using the PDF.

## 5. A Relational Powder Diffraction File

There is much more information in the PDF (and Crystal Data, which uses the same format, NBS*AIDS83) than is used directly in traditional methods of phase identification. In searching for the answer to a problem, all of this information is potentially useful. Several years ago, we adapted relational-database technology to search these databases in unconventional ways. The sort of question you’d like to answer is: “What green copper-containing compounds have one of their 10 strongest lines between 2.58 < *d* < 2.62 Å?” (35-502, (Cu,Zn)_2_CO_3_(OH)_2_ is one.)

Rather than invent our own algorithms, we chose to use a commercial relational database system. We happened to have and use the VAX-based System 1032, but know that other programs (particularly Oracle and Paradox) have been used successfully in similar applications. The major problem in implementing a relational PDF is that relational database systems work on “tables”—matrices of data, with well-defined rows and columns. The NBS*AIDS83 format (Table 2) is not “relational database friendly,” and needs to be converted into something which can be loaded into a relational database system.

Before the data are converted, there needs to be a plan for the conversion—another way of saying that a data base structure needs to be designed. Our original versions contained virtually all of the fields in the AIDS format (including the editorial codes!). With actual use, we found that only some of the information was useful in materials identification, and we reduced the content of the final database.

Design of a relational database is a non-trivial task. The needs and wants of both the users and the database builders must be considered. Since I was to be the principal user, this task was somewhat easier, and the database could be designed to fit my thought patterns. Because of these preferences and ease of building, a complex database design was derived. This consists of five joined datasets, linked through the common field of the PDF (or CD) number. We used the existing information, and created some new fields. The final database contains text, integer, floating point, vector, and logical fields. The five datasets are summarized in Table 3. Only some of the fields are indexed.

FORTRAN programs were written to convert the NBS*AIDS83 format into one suitable for building a database. The strategy followed was crude, but effective. The PDF is a large file (the Set 44 release was 154 megabytes). It turned out to be necessary as well as desirable to break up this large file into individual sets—to minimize scratch space during loading, but also to be able to edit the file to correct errors. In our initial trials we found several cases of illegal data in particular fields. There were a very few cases in which the data present in the PDF did not correspond to the specified format. The AIDS-format files were read once, and an intermediate file, containing only the card number, card type, and record number, was generated. This file was used to reread the AIDS-format data into the main conversion program. In this program, there is one subroutine to process each record type. It creates the input files for the database building. The loading and indexing tools of the database system were used to build the final database. The whole process requires about 24 hours of CPU time on a MicroVAX II.

The toughest part of the task of converting the data was parsing the formulas and generating the elemental bitmaps. Very useful quantities generated during the conversion are the *element count* (the number of different elements present in the formula) and the *sequence number* of an individual line in the powder pattern. The observed lines were sorted in order of decreasing intensity, and their ordinal rank stored in the database.

Each database system has its own syntax. It is sometimes cumbersome to obtain the desired information, and multiple queries may need to be combined, but it is generally possible to extract the answer one desires. Output routines for convenient display of the PDF data were written. We were even able to “trick” the database system into generating a graphical display (“stick pattern”) of the powder pattern by generating a bar graph. All of the source code for the conversion programs is available from the author at no charge.

**Fig. 8:**
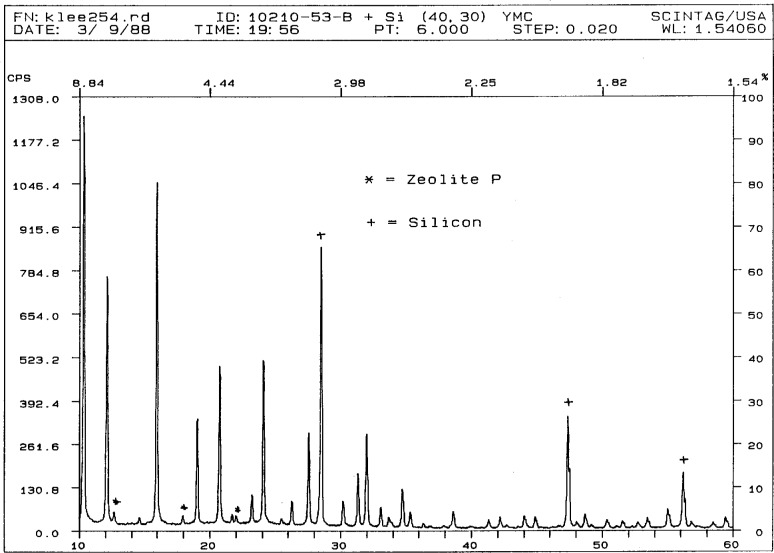
The powder diffraction pattern of a steamed faujasite (zeolite Y). The weak peaks indicated by asterisks indicate the presence of a trace of zeolite P impurity.

A particularly interesting example of the use of the relational PDF is a problem concerning a steamed dealuminated zeolite Y. Three extra peaks were present in the powder pattern of the steamed zeolite (Fig. 8), and there was concern that a condensed silica phase had been generated. The usual Hanawalt search techniques did not yield any plausible phases to account for these weak peaks. The relational PDF was used to obtain an identification.

The selection set was limited to phases containing Si, Al, and O. The individual lines in the patterns of these phases were searched for lines occurring in narrow windows about each of the three observed lines. The small number of phases which contained all three of these lines turned out to correspond to various forms of zeolite P, a common coproduct in the synthesis of zeolite Y and a reasonable impurity phase in a product derived from commercial material. The observed lines are the 2nd, 3rd, and 5th strongest lines in the pattern. The other strong lines are obscured by the lines of zeolite Y.

A relational database provides the flexibility to search the data in unanticipated ways. It turns out to be a powerful tool for editorial applications. It is easy to spot the “garbage” and missing data. The disadvantages of applying relational technology to the PDF and NIST CD are that there is a lot of missing data, and that the syntax is not controlled. Before the Zeolite and Molecular Sieve Index was developed, it was very difficult to identify all of the zeolites in the PDF. The notation “zeolite” or “molecular sieve” was contained sometimes in the comments fields, sometimes in the structure type field, sometimes in other places, or often not listed at all.

This relational PDF has been a useful tool for several years. As the PDF (PC-PDF and PCPDFWIN) has developed, many of the capabilities I sought have been implemented. The fully-relational system is still useful in special cases. The ICDD hopes to incorporate relational technology in future database designs.

Relational technology is not new. It is interesting to ask what use can be made of more-recent advances in database technology. Much is made today of “object oriented” databases. A powder pattern could be considered a graphical object. It is intriguing to ask whether one could make use of object oriented systems in phase identification. Could considering a powder pattern as a graphical object yield new measures of similarity?

The crystallographic databases are large complex datasets. It is important that we keep abreast of advances in database technology, so that they can be applied when suitable. None of the database suppliers have the resources to invent all of the necessary tools, so they need to use what is available. It is easy to imagine that at sometime in the future these datasets could be supplied in formats suitable for loading into the user’s database system of choice.

## Figures and Tables

**Table 1:** Atom coordinates and displacement coefficients of VOP_2_O_6_ Space Group I2/a, *a* = 12.170(2), *b* = 4.1998(13), *c* = 9.573(2) Å, *β* = 92.83(2)°, *Z* = 4

Atomic coordinates (×10^4^) and equivalent isotropic displacement coefficients (Å^2^×10^3^)				
Atom	*x*	*y*	*z*	*U*iso
V	1/4	4993(1)	1/2	7(1)
P	787(1)	7542(1)	7311(1)	7(1)
O1	1/4	1203(3)	1/2	14(1)
O2	1164(1)	5985(2)	6025(1)	11(1)
O3	1605(1)	5849(2)	3263(1)	11(1)
O4	136(1)	5023(2)	8201(1)	10(1)

Equivalent isotropic *U* defined as one third of the trace of the orthogonalized *U_ij_* tensor.				

Anisotropic displacement coefficients (Å^2^×10^3^)						
Atom	*U*11	*U*22	*U*33	*U*23	*U*13	*U*12
V(1)	6(1)	8(1)	6(1)	0	0(1)	0
P(1)	5(1)	8(1)	6(1)	−1(1)	1(1)	0(1)
O(1)	16(1)	10(1)	15(1)	0	0(1)	0
O(2)	9(1)	15(1)	10(1)	−3(1)	3(1)	0(1)
O(3)	10(1)	14(1)	10(1)	1(1)	−2(1)	2(1)
O(4)	10(1)	10(1)	9(1)	1(1)	0(1)	−2(1)

The anisotropic displacement exponent takes the form: −2π^2^(*h*^2^a^*2^*U*_11_ + … + 2hka^*^b^*^U_12_).						

**Table 2:** NBS*AIDS83-format of PDF entry 44-430, NaAlO_2_·5/4H_2_O

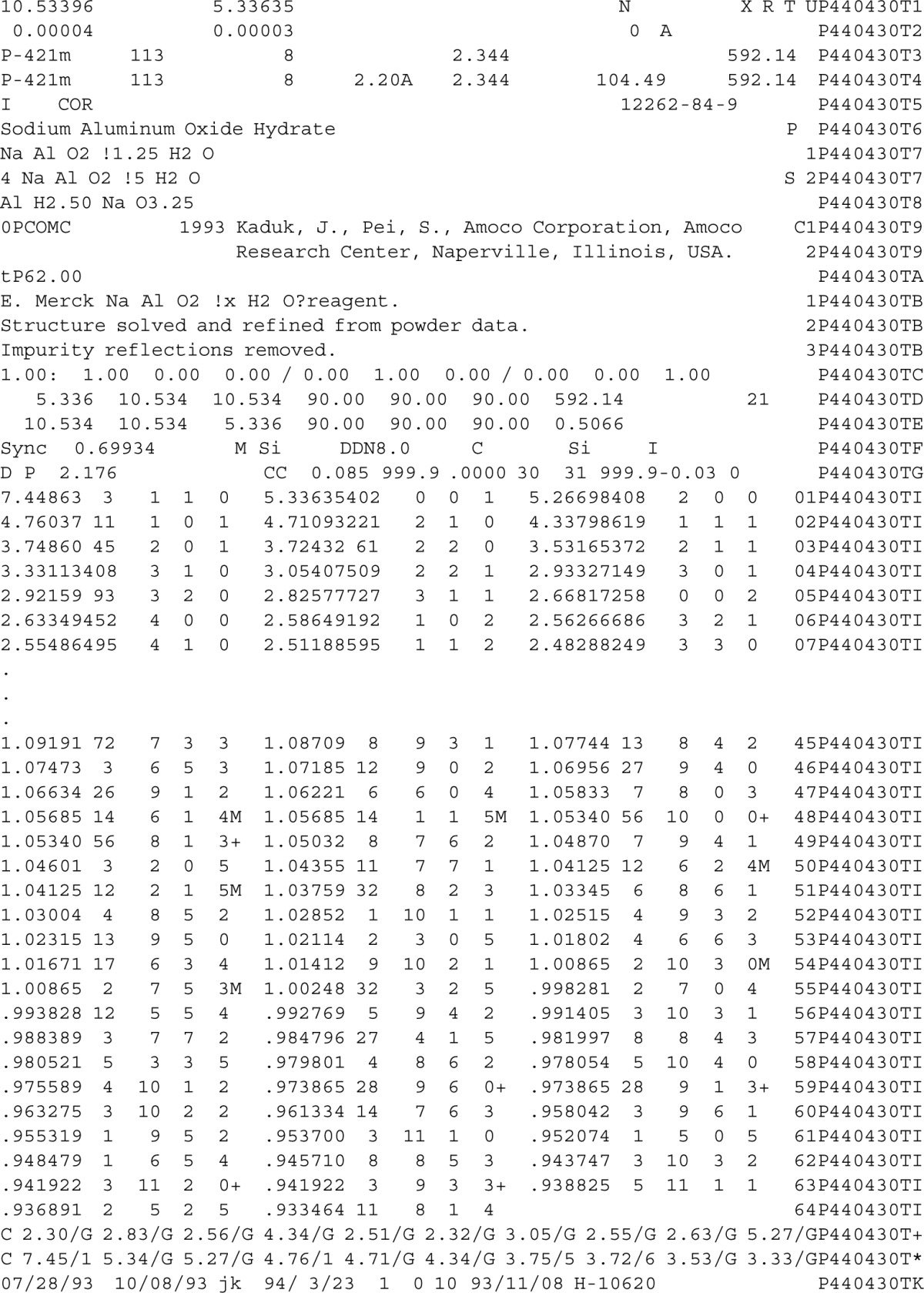

**Table 3:** Structure and fields in the relational powder diffraction file

**STUFF**—single record/entry				
*Card*	Name	Formula	Em. Form.	CASRN
Phase	S. Type	Quality	RIR	A.M.Wt.
Coden	Volume	Page	Year	Authors
A. Sp. Gr.	Sp. Gr. #	A. Z	A. D_m_	A. D_c_
CD Sp. Gr.	CD SG #	CD Z	CD APD	CD D_x_
Radiation	Λ	Int. Std.	R Factor	SS FOM
DW FOM	C_σ_	Agreem.	Avg∆2θ	
**CELLS**—single record/entry				
*Card*	A. Cell(6)	Avg. Err.	A. Vol.	σCell(6)
R. Cell(6)	RF #	RC Vol.	CDCell(6)	CD Vol.
**ELEMENTS**—single record/entry—an “elemental bitmap”				
*Card*	***El. Count***	*Individual Elements*		
Groups	*Periods*			
**COMMENTS**—multiple records/entry				
*Card*	Comment	*Comment Code*		
**PATTERN**—multiple records/entry				
*Card*	*d*	*I*	***Sequence***	
h	k	l		

Italicized items are indexed. Fields in boldface were created during the database building process, and are not present in the original NBS*AIDS83-format database.

## References

[1] Allen FH, Kennard O (1993). Chem Design Automation News.

[2] Bergerhoff G, Hundt R, Sievers R, Brown ID (1983). J Chem Inform Comput Sci.

[b3-j3kadu] 3Canada Institute for Scientific and Technical Information, National Research Council, Ottawa, Ontario, Canada K1A OS2; cansnd@info.cisti.nrc.ca.

[b4-j3kadu] 4Protein Data Bank, Chemistry Department, Brookhaven National Laboratory, P.O. Box 5000, Upton NY 11973-5000; pdb@bnl.gov.

[5] Berman HM, Olson WK, Beveridge DL, Westbrook J (1992). Biophys J.

[b6-j3kadu] 6NIST Crystal and Electron Diffraction Data Center, National Institute of Standards and Technology, Gaithersburg, MD 20899-0001.

[b7-j3kadu] 7International Centre for Diffraction Data, 12 Campus Boulevard, Newtown Square PA 19073-3273; info@icdd.com.

[8] Shklover VE, Struchkov YuT, Ganyushin AV (1985). Zh Strukt Khim.

[9] Derissen JL (1974). Acta Cryst B.

[10] Bailey M, Brown CJ (1967). Acta Cryst.

[11] Brown CJ (1984). Acta Cryst C.

[12] Davey RJ, Maginn SJ, Andrews SJ, Buckley AM, Cottier D, Dempsey P, Rout JE, Stanley DR, Taylor A (1993). Nature.

[13] Davey RJ, Maginn SJ, Andrews SJ, Black SN, Buckley AM, Cottier D, Dempsey P, Plowman R, Rout JE, Stanley DR, Taylor A (1994). J Chem Soc Faraday Trans.

[14] Visser JW (1969). J Appl Crystallogr.

[15] Ward DL, Luehrs DC (1983). Acta Cryst C.

[16] Robl C, Hentschel S (1991). Mater Res Bull.

[17] (1967). Proc 10th Internat Conf Coord Chem Tokyo.

[18] Tanaka Y, Fukunaga J, Setoguchi M, Higashi T, Ihara M (1982). J Ceram Soc Jpn.

[19] Howie R, West A (1977). Acta Cryst B.

[20] Kaduk JA, Satek LC

[b21-j3kadu] 21J. A. Kaduk and S. T. McKenna, unpublished results.

[22] Satek LC, Kaduk JA, McMahon PE, Pascoe WE (1992). Catalysis of Organic Reactions.

[23] Richter L (1977). Synthese und Strukturuntersuchungen von Eisen- und Kupfer-Aluminum-Boraten.

[b24-j3kadu] 24L. C. Satek, J. A. Hriljac, R. D. Brown, J. A. Kaduk, and A. K. Cheetham, unpublished results.

[25] Kaduk JA, Faber J, Pei S

[26] Brown ID, Altermatt D (1985). Acta Cryst B.

[b27-j3kadu] 27G. W. Zajac, J. Faber, and S. Pei, unpublished results.

[28] Wells AF (1938). Zeit Krist.

[29] Schäfer H, Weise U, Rinke K, Brendel K (1967). Angew Chem Int Ed.

[30] Soulen JR, Chappell WH (1965). J Phys Chem.

[31] Wells AF (1947). J Chem Soc.

[32] Gorbunova YE, Linde SA (1979). Dokl Akad Nauk SSSR.

[33] Middlemiss NC (1978). PhD Thesis.

[34] Thompson MR, Hess AC, Nicholas JB, White JC, Anchell J, Ebner JR, Cortés Corberán V, Vic Bellón S (1994). New Developments in Selective Oxidation.

[35] Nguyen PT, Hoffman RD, Sleight AW (1995). Mater Res Bull.

[36] Krasnikov VV, Konstant ZA (1979). Isv Akad Nauk SSSR Neorg Mater.

[37] Bordes E, Courtine P (1979). J Catal.

[38] Krasnikov VV, Konstant ZA (1983). Kristallografiya.

[39] Linde SA, Gorbunova YuE (1983). Zh Neorg Khim.

[40] Krasnikov VV, Konstants Z, Gedrovics J, Ozolins G, Zviedre I, Actina L (1977). Latv PSR Zinat Akad Vestis Kim Ser.

[41] Tofield BC, Pasteur GA, Sherwood RC (1975). J Chem Soc Dalton Trans.

